# The effect of pandemic as a trigger for first episode bipolar disorders

**DOI:** 10.1192/j.eurpsy.2023.1487

**Published:** 2023-07-19

**Authors:** S. Cakir

**Affiliations:** Psychiatry, MoodART Clinic, Istanbul, Türkiye

## Abstract

**Introduction:**

The traumatic effects of COVID-19 pandemic is well studied in community and fragile groups. The association between COVID-19 infection and development of severe mood disorders have not well studied.

**Objectives:**

Nonetheless the casual relation or stressor effects of Covid-19 pandemic on chronic psychiatric illness is not known yet. The present study is aimed to investigate the effects of pandemic as a triggering factor in first episode Bipolar disorder (BD) patients that onset after pandemic.

**Methods:**

The study included a total sample of 55 patients diagnosed with first episode BD according to DSM-5 criteria.

The two groups of patients that illness onset was before (BP)and after pandemic (AP), were investigated and compared for psychopathology and life evet stressors. Impact of Event Scale-Revised (IES-R) for PTSD symptoms, Generalize Anxiety Disorder scale for anxiety symptoms, The Montgomery–Åsberg Depression Rating Scale (MADRS) to examine depressive symptoms; and Young Mania rating Scale (YMRS) for manic symptoms, Brief Psychiatric Rating Scale-18 (BPRS) was used for psychotic symptoms.

**Results:**

The statistical analyses were performed using the Statistical Package for Social Science, version 26. Thirty-five patients that illness onset before pandemic and 20 patients that illness onset after pandemic were compared.

The significant differences were come up in some clinical features between the two groups. The alcohol and substance abuse were higher in AP group. The severity of psychotic and manic symptoms were higher in AP group. The hospitalization was higher in AP group. The number of stressor events was higher and PTSD symptoms was more severe in the AP group also.

**Conclusions:**

The effects of Covid-19 pandemic seems have a triggering role in onset of first episode BD. This effect whether cause biological or psychological stress in onset of illness is not known yet. The casual phenomenon of Covid-19 pandemic should be investigated for chronic psychiatric illness as BD in future studies.

**Disclosure of Interest:**

None Declared

